# Evaluation of different probiotics on growth, body composition, antioxidant capacity, and histoarchitecture of *Mugil capito*

**DOI:** 10.1038/s41598-024-57489-x

**Published:** 2024-03-28

**Authors:** Akram Ismael Shehata, Ali A. Soliman, Hamada A. Ahmed, Mahmoud S. Gewaily, Asem A. Amer, Mustafa Shukry, Hany M. R. Abdel-Latif

**Affiliations:** 1 Department of Animal and Fish Production, Faculty of Agriculture (Saba Basha), Alexandria University, Alexandria, 21531 Egypt; 2 National Institute of Oceanography and Fisheries (NIOF), Alexandria, Egypt; 3 Department of Nutrition and Veterinary Clinical Nutrition, Faculty of Veterinary Medicine, Damanhour University, Damanhour, 22511 Egypt; 4 Department of Anatomy and Embryology, Faculty of Veterinary Medicine, Kafrelsheikh University, Kafrelsheikh, 33516 Egypt; 5 Department of Fish Nutrition and Feed Technology, Central Laboratory for Aquaculture Research, Agricultural Research Center, Abbassa, Abo-Hammad, Sharqia 44662 Egypt; 6 Department of Physiology, Faculty of Veterinary Medicine, Kafrelsheikh University, Kafrelsheikh, 33516 Egypt; 7 Department of Poultry and Fish Diseases, Faculty of Veterinary Medicine, Alexandria University, Alexandria, 22758 Egypt

**Keywords:** Probiotics, Growth, Antioxidant, Hepatic health, Mullets, Biochemistry, Immunology, Physiology

## Abstract

We investigated the dietary effects of the single application of *Saccharomyces cerevisiae*, *Lactobacillus bulgaricus*, and their combination on growth, proximate composition of whole fish body, antioxidant defense, and histoarchitecture of hapa-reared *Mugil capito*. Healthy fish (Fish weighed = 10.30 ± 0.10 g at first) were randomly allocated into 4 equal groups, each with three replicates. These groups were designed as follows: (1) a group fed a basal diet without probiotics (control), (2) a group fed a diet containing *S. cerevisiae* (4 g/kg diet), (3) a group fed a diet containing *L. bulgaricus* (2 g/kg diet), and (4) the last group fed a diet containing a combination of both, all for a duration of 60 days. Probiotic-treated groups showed significantly better growth and nutrition utilization than the control group. Significant differences were observed in the crude fat and crude protein contents among the groups, with the combination group exhibiting the highest levels. However, there were no significant variations in ash content across all groups. The highest hepatic antioxidant capacity (superoxide dismutase (SOD), catalase (CAT), and glutathione peroxidase (GPX) enzyme activities) was observed in the combination group. Thiobarbituric acid reactive substance (TBARS) concentrations were decreased significantly in all probiotic groups, suggesting improved oxidative stress resilience in these groups. The histomorphological analysis of the hepatopancreatic tissues revealed well-arranged parenchyma, increased glycogen storage, and melanomacrophage centers in probiotic-treated groups, particularly the combined probiotics group. Furthermore, the probiotic supplementation improved the histoarchitecture of the intestinal villi compared to the control group. To put it briefly, combined dietary administration of these probiotics improved growth, body composition, antioxidant defenses, and hepatic and intestinal health in hapa-reared *M. capito*, highlighting their promising role in promoting welfare and productivity.

## Introduction

The aquaculture industry plays a fundamental role in meeting the growing global demand for food. The intensification of fish farming practices has led to various challenges, including disease outbreaks and environmental concerns^1,2^. As a result, there is an increasing need for elaborating effective approaches to enhance health and productivity^3,4^. Probiotics are beneficial microorganisms that can be used as a promising solution in this regard^5–8^. Multiple reports indicate that the addition of probiotics as a supplement can enhance fish growth, optimize feed utilization, boost immune responses, and improve stress resistance^4,9–11^.

The probiotic yeast, *Saccharomyces cerevisiae,* and the probiotic bacteria, *Lactobacillus bulgaricus* are widely studied for their beneficial effects on the health of various animal species^12–15^. *S. cerevisiae* is the most frequently used probiotic yeast in aquaculture and is recognized for its ability to enhance nutrient utilization and stimulate fish’s immune system^16^. Several authors have hypothesized that the cellular components of this yeast such as β-glucan, mannan oligosaccharides, glucooligosaccharides, and enzymes exhibited excellent growth-promoting and immunological roles for fish^17–20^. *L. bulgaricus*, a well-known probiotic bacterium commonly associated with yogurt production, has recently gained attention for its potential application in fish farming and aquaculture industry^21,22^. Moreover, reports showed that *L. bulgaricus* had a potential role in improving the overall well-being and productivity of fish in aquaculture systems^14,23^.

Multi (multiple) species probiotics can be broadly characterized as a combination or blend of two or more species, typically offering greater advantages to the host compared to single-strain probiotics^24,25^. While the individual effects of *S. cerevisiae* and *L. bulgaricus* have been considerably studied in aquaculture, limited research has explored their combined application and their effects on fish health and performance. Furthermore, it is pertinent to delve deeper into the potential interactions that may occur between various probiotics and assess whether these interactions could have detrimental or beneficial effects on fish health. Such exploration could propose valuable perceptions for the development of effective and sustainable aquaculture.

*Mugil capito* is an important fish species with high nutritional value^26^. However, similar to other farmed fish, this fish is susceptible to a range of stressors within aquaculture environments. Stressors as suboptimal nutrition have the potential to undermine the growth, immune function, and overall performance of farmed fish like *M. capito*^27–29^. Therefore, exploring effective dietary strategies to enhance the growth, health, and antioxidant capacity of mullet is of great importance for maintaining sustainable aquaculture. Hence, this study aims to investigate the dietary effects of *S. cerevisiae*, *L. bulgaricus*, and their mixture on the overall performances of *M. capito*, focusing on their effects on growth, proximate composition of whole fish body, antioxidant capacity, and intestinal and hepatic histoarchitecture.

## Materials and methods

### Ethical statement

The study’s ethical considerations were approved by the Faculty of Veterinary Medicine, Alexandria’s Local Experimental Animal Care Committee (Ethical Approval code: AU-013/2023/09/11-3R/4P/256). Additionally, all study techniques adhered to the ARRIVE criteria, version 2.0^30^, ensuring that the study procedures followed accepted ethical guidelines and safeguarded the welfare of the fish subjects.

### Fish acquisition and acclimation

One hundred and twenty healthy *M. capito* were sourced from a private farm located in the district of Kafr Elsheikh, Egypt. These fish were housed and nurtured in four 500-L fiberglass aquariums, each equipped with efficient aeration systems to maintain water quality. To ensure their successful acclimatization to the new environment, fish were allowed to acclimate in these tanks for 2 weeks. During this time, fish were provided with ad libitum feeding, using a nutritionally balanced commercial diet sourced from Aller Aqua Co., Egypt.

### Probiotics used and diet preparation

The *S. cerevisiae* cell wall extract (Megamos®) utilized in this research was commercially purchased from Biobridtech (USA) by a Local Agent supplier. It was added to the baseline diet at a rate of 4 g/kg diet, as recommended in the prior studies conducted by Islam et al.^31^ and Fadl et al.^32^. *Lactobacillus bulgaricus* (Batch No. EL1010322) is a freeze-dried powder purchased commercially from APEX BIOTECHNOL in Indore, Madhya Pradesh, India, via Free Trade Egypt Company in Alexandria, Egypt. This strain of *L. bulgaricus* is marketed as a probiotic food-grade dietary supplement, boasting a potency of 21.5 × 10^9^ CFU/g, which underscores its high viability and total cell count. It was incorporated into the basal diet at a supplementation dose of 2 g/kg diet. The probiotic strains were maintained at − 20 °C until it was needed for experimentation. To assess the comparative effectiveness of single-strain and their mixture in enhancing the growth and health status of *M. capito*, we designed four distinct diets. The control group received only the baseline diet without any additional supplements (refer to Table S1 in the Supplemental Material). The second diet involved the supplementation of the basal diet with *S. cerevisiae* (4 g/kg diet). For the third diet, *L. bulgaricus* (2 g/kg diet) was incorporated into the basal diet. The fourth diet combined both supplements (*S. cerevisiae*, 4 g/kg diet + *L. bulgaricus*, 2 g/kg diet). The test probiotic doses were mixed into a powdered nutrient base to ensure uniform distribution. Water was added to the feed ingredients to form a workable paste, which was then processed through a food pelleting machine to create uniform 2-mm pellets. These pellets were air-dried and stored in airtight plastic bags at − 20 °C until required. The diets were formulated to be isonitrogenous and isocaloric, meeting the nutritional requirements for rearing fish as per the guidelines of NRC^33^.

### Experimental design and setup

Following acclimation, fish weighing 10.30 ± 0.10 g were randomly allocated to triplicate experimental groups and placed in twelve hapas, each containing 10 fish. These hapas were located in cement raceway ponds at the Baltim Research Station, National Institute of Oceanography and Fisheries, Egypt. During the feeding trial, fish were hand-fed daily with the formulated test diets until they exhibited obvious satiety. The feeding rate was maintained at 5% of the fish’s wet body weight for 60 days. Bi-weekly weight measurements were performed to adjust the diet quantities, while any residual feed and feces were removed daily to prevent the accumulation of unionized ammonia. Each hapa was equipped with continuous aeration to maintain optimal conditions for fish growth. The lighting schedule followed a 12-h light and 12-h darkness cycle (12L:12D). Dissolved oxygen (DO) levels (mg/L), temperature (°C), pH value, nitrite (NO_2_) and unionized ammonia (NH_3_) levels were measured. Throughout the experiment, water measurements were in the range temperature (28.5–30.5 °C), DO (6.90 ± 0.40 mg/L), pH (7.80–8.10), as well as NO_2_ and NH_3_ were 0.03 ± 0.01 mg/L, and 0.05 ± 0.01 mg/L, respectively. Water salinity was measured by a salinometer, and its value range was 5–6 ppt. These parameters were monitored weekly, with no significant deviations observed during the experimental period. These stable conditions were deemed suitable for the successful rearing of mullet in this study.

### Samples collection

After the feeding trial, the basal and test diets were withheld from the fish in all hapas for one day before sampling. Initially, the total number of fish per hapa, individual body weight, and total weight in each hapa were determined. Clove oil at a concentration of 20 mg/L was used for fish anesthesia. Nine fish (three from each hapa) were randomly selected from each group, euthanized with an overdose of clove oil (40 mg/L), and their livers and intestines were then aseptically extracted to collect samples. The samples were immediately rinsed with sterile cold phosphate-buffered saline (PBS) solution (pH ~ 7.4) and fixed in a 10% buffered formalin solution for 2 days to facilitate histological examination. Additionally, a set of liver samples (nine per group) were taken for the preparation of tissue homogenates to assess hepatic oxidative stress biomarkers. Another set of 3 fish per group was sampled to evaluate the chemical composition of the fish’s whole body.

#### Growth parameters and measurements

The following formulas were utilized to calculate growth, feed utilization parameters, and survival rate.

Weight gain (WG):

$${\text{WG }}\left( {\text{g}} \right) \, = {\text{ Final body weight }}\left( {{\text{FBW}}} \right) - {\text{Initial body weight }}\left( {{\text{IBW}}} \right).$$

Specific growth rate (SGR):

$${\text{SGR}}(\% /{\text{d}}) = \, \left[ {\left( {{\text{Ln }}\left( {{\text{FBW}}} \right) \, {-}{\text{ Ln }}\left( {{\text{IBW}}} \right)} \right)/{\text{days}}} \right] \, \times { 1}00,$$

where “Ln” represents the natural logarithm and “days” is the number of days over which the growth occurred.

Feed intake (FI):

$${\text{FI }}\left( {{\text{g}}/{\text{fish}}} \right) \, = {\text{ Total feed offered }} - {\text{ Total feed remaining}}.$$

Feed conversion ratio (FCR):

$${\text{FCR }} = {\text{ Total feed consumed}}/{\text{Weight gain }}\left( {{\text{WG}}} \right).$$

Protein efficiency ratio (PER):

$${\text{PER }} = {\text{ Weight gain }}\left( {{\text{WG}}} \right)/{\text{Total protein consumed}}.$$

Protein productive value (PPV):

$${\text{PPV }}\left( \% \right) \, = \, \left( {{\text{Weight gain }}\left( {{\text{WG}}} \right) \, \times { 1}00} \right)/{\text{Total protein consumed}}.$$

Energy retention (ER):

$${\text{ER }} = {\text{ Energy content of fish at the end }} - {\text{ Energy content of fish at the start}}.$$

Survival rate (SR):

$${\text{SR }}\left( \% \right) \, = \, \left( {{\text{Number of survived fish}}/{\text{Initial number of fish}}} \right) \, \times { 1}00.$$

#### Chemical composition analysis

The moisture, crude protein (CP), crude lipids (CL), and ash content were conducted following the guidelines outlined by AOAC^34^.

#### Hepatic antioxidant capacity analysis

Utilizing a Teflon-coated mechanical homogenizer, 0.1 g of hepatic tissue was homogenized in 0.9 mL of PBS solution. The samples were subsequently centrifuged for 15 min at 3500×*g* to obtain the supernatant. Hepatic oxidative stress biomarkers, including SOD, CAT, and GPX enzyme activities, were assessed using commercial ELISA kits (SOD kit: CSB-E15929Fh, CAT kit: CSB-E15928Fh, GPX kit: CSB-E15930Fh) obtained from Cusabio Biotech Company, Ltd. (Wuhan, China), following the guidelines provided by the manufacturer. The concentration of hepatic Malondialdehyde (MDA) was determined using commercial kits from Biodiagnostic Co. (Giza, Egypt). MDA concentrations, indicative of lipid peroxidation, in the test samples were determined using a spectrophotometric technique as detailed by Ke et al.^35^ based on the assessment of thiobarbituric acid reactive substance (TBARS) in fish tissues. Furthermore, following the methodology outlined by Benzie and Strain^36^, an antioxidant power experiment was conducted at an optical density (OD) of 593 nm.

#### Intestinal and hepatic histoarchitecture

The livers and intestines preserved in formalin were processed using the paraffin embedding technique, following the guidelines outlined in Bancroft and Gamble^37^. Tissue slices were stained with hematoxylin and eosin, and observed using an Olympus BX50/BXFLA microscope (Japan).

### Statistical analysis

The data were analyzed using one-way ANOVA followed by post hoc analysis with Duncan’s Multiple Range Test. The findings were presented as mean values with standard error of the mean (SEM). Statistical significance was considered when *p* < 0.05. SPSS software was utilized for the statistical analysis.

## Results

### Growth performance, nutrient utilization, and survival rate of *M. capito*

In Table 1, several indicators exhibited notable differences among the treatments. The combination treatment group exhibited the highest FBW than the rest of the treatments. This trend was consistent with WG, SGR, and FI, where the combination group outperformed the other groups, indicating significant differences (*p* < 0.05). Additionally, the PER was significantly higher in the combination group (*p* < 0.05), compared to the control group. Furthermore, the PPV demonstrated a similar trend, with the combination group surpassing other groups significantly (*p* < 0.05). Finally, the highest protein efficiency was observed in the combination group, evident from the elevated E. value compared to other treatments. Notably, SR was consistent across all groups, remaining at 100%.

**Table 1:** Impacts of probiotic supplements (*S. cerevisiae*, *L. bulgaricus*, and their combination) on growth, nutrient utilization, and survival of *M. capito* over a 60-day trial.

Variables	Experimental groups				*p*-value
	Control	*S. cerevisiae*	*L. bulgaricus*	Combination***	
IBW (g/fish)	10.35 ± 0.06	10.44 ± 0.11	10.30 ± 0.32	10.10 ± 0.09	N/A
FBW (g/fish)	19.11 ± 0.19^d^	26.44 ± 0.11^c^	27.26 ± 0.00^b^	28.29 ± 0.02^a^	0.001
WG (g/fish)	8.76 ± 0.13^d^	16.00 ± 0.22^c^	16.96 ± 0.32^b^	18.19 ± 0.11^a^	0.001
SGR (%/d)	1.02 ± 0.01^c^	1.55 ± 0.02^b^	1.62 ± 0.05^b^	1.72 ± 0.01^a^	0.001
FI (g feed/fish)	24.75 ± 0.14^b^	30.75 ± 0.43^a^	32.00 ± 0.29^a^	30.85 ± 0.72^a^	0.001
FCR	2.83 ± 0.06^a^	1.92 ± 0.00^b^	1.89 ± 0.02^b^	1.70 ± 0.05^c^	0.001
PER	1.15 ± 0.02^c^	1.71 ± 0.00^b^	1.71 ± 0.02^b^	1.89 ± 0.05^a^	0.001
PPV (%)	13.65 ± 0.18^b^	15.20 ± 0.57^a^	15.34 ± 0.23^a^	15.97 ± 0.46^a^	0.018
ER	14.18 ± 0.79^c^	18.51 ± 1.11^bc^	21.55 ± 2.34^b^	30.62 ± 2.46^a^	0.001
SR (%)	100	100	100	100	N/A

All values are presented as mean ± SE, n = 3.

*IBW* initial body weight, *FBW* final body weight, *WG* weight gain, *SGR* specific growth rate, *FI* feed intake, *FCR* feed conversion ratio, *PER* protein efficiency ratio, *PPV* protein protective value, *ER* energy retention, *SR* survival rate.

The alphabetical superscripts within the values denote significant differences (*p* < 0.05) between various treatments within each row.

***Combination = *S. cerevisiae* + *L. bulgaricus.*

### Proximate body composition of *M. capito*

The results indicate significant differences among the treatment groups for various parameters in Table 2. Moisture content (%) was highest in the control group, while the combination of *S. cerevisiae* and *L. bulgaricus* resulted in the lowest moisture content. CP showed significant variation (*p* < 0.05), with the highest value observed in the combination group compared to the control. Similarly, CL content was significantly different across treatments (*p* < 0.05), with the combination group exhibiting the highest value. In contrast, ash content showed no significant differences among the groups (*p* > 0.05).

**Table 2:** Impacts of probiotic supplements (*S. cerevisiae*, *L. bulgaricus*, and their combination) on proximate body composition of *M. capito* over a 60-day trial.

Variables	Experimental groups				*p*-value
	Control	*S. cerevisiae*	*L. bulgaricus*	Combination***	
Moisture (%)	71.25 ± 0.35^a^	69.08 ± 0.75^b^	69.64 ± 0.33^ab^	68.61 ± 0.62^b^	0.040
CP (%)	15.45 ± 0.18^a^	16.76 ± 0.56^ab^	16.73 ± 0.25^ab^	17.40 ± 0.45^b^	0.041
CL (%)	6.96 ± 0.28^c^	7.39 ± 0.24^bc^	7.92 ± 0.26^b^	8.80 ± 0.25^a^	0.005
Ash (%)	4.93 ± 0.17	5.00 ± 0.29	5.20 ± 0.13	4.59 ± 0.21	0.285

All values are presented as mean ± SE, n = 3.

*CP* crude protein, *CL* crude lipid.

The alphabetical superscripts within the values denote significant differences (*p* < 0.05) between various treatments within each row.

***Combination = *S. cerevisiae* + *L. bulgaricus.*

### Hepatic antioxidant activity of *M. capito*

The results of the study demonstrate significant variations in antioxidant enzyme activity and oxidative stress biomarkers among the tested groups, as illustrated in Table 3. It was found that *S. cerevisiae* and *L. bulgaricus* individually showed increased SOD, CAT, and GPX activities in comparison to the control group. The combination group exhibited the highest antioxidant enzymatic activities (*p* < 0.05). Conversely, TBARS assay results, an indicator of oxidative stress, displayed the opposite trend. The control group had the highest TBARS levels, while *S. cerevisiae*, *L. bulgaricus*, and the combination group exhibited lower TBARS levels, with the combination group showing the most pronounced reduction (*p* < 0.05).

**Table 3:** Impacts of probiotic supplements (*S. cerevisiae*, *L. bulgaricus*, and their combination) on hepatic antioxidant activity of *M. capito* over a 60-day trial.

Variables	Experimental groups				*p*-value
	Control	*S. cerevisiae*	*L. bulgaricus*	Combination***	
SOD (U/mg protein)	31.10 ± 0.79^c^	38.42 ± 0.90^b^	39.10 ± 0.90^b^	45.02 ± 2.77^a^	0.002
CAT (U/mg protein)	11.61 ± 0.20^c^	14.13 ± 0.33^b^	14.44 ± 0.39^b^	16.65 ± 1.09^a^	0.003
GPX (U/mg protein)	32.12 ± 0.35^c^	46.10 ± 1.08^b^	46.92 ± 1.08^b^	54.02 ± 3.32^a^	0.001
TBARS (nmol/mg protein)	42.26 ± 0.54^a^	36.02 ± 6.66^b^	36.83 ± 3.23^b^	24.46 ± 3.08^c^	0.001

All values are presented as mean ± SE, n = 3.

*SOD* superoxide dismutase, *CAT* catalase, *GPX* glutathione peroxidase, *TBARS* thiobarbituric acid reactive substance.

The alphabetical superscripts within the values denote significant differences (*p* < 0.05) between various treatments within each row.

***Combination = *S. cerevisiae* + *L. bulgaricus*.

### Histological observations of the intestinal and hepatic in *M. capito*

**Figure 1:**
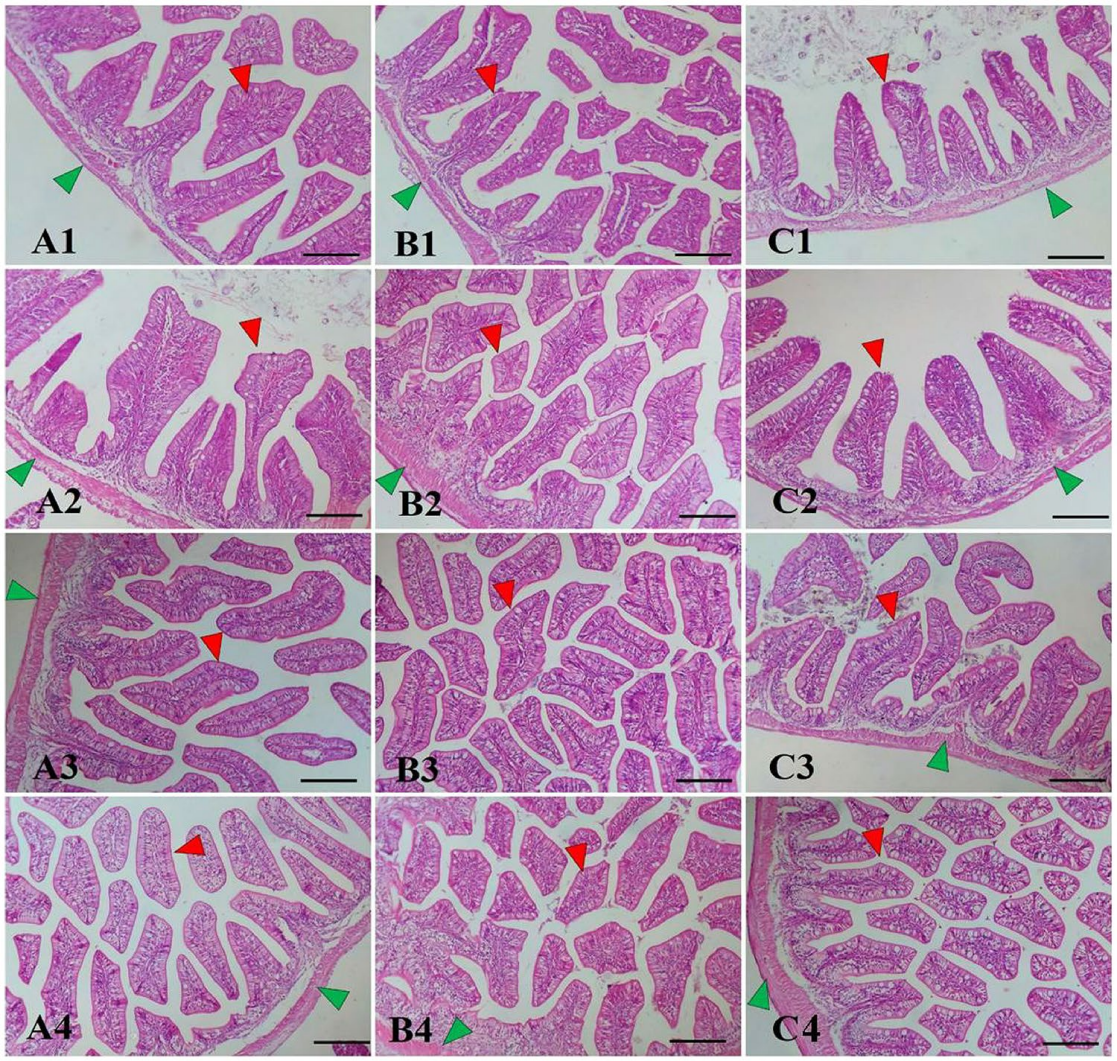
Photomicrograph showing the histological structure of anterior (**A1–A4**), middle (**B1–B4**), and posterior (**C1–C4**) intestine of *M. capito*. The control group (**A1–C1**), *S. cerevisiae* (**A2–C2**), *L. bulgaricus* (**A3–C3**), and their combination (**A4–C4**). The intestinal villi (red arrowhead) and wall (green arrowhead) demonstrated apparent improvement by supplemented *S. cerevisiae* and/or *L. bulgaricus* levels. Stain H&E. Bar = 100 µm.

The histological structure of *M. capito* intestine showed intact structures of the intestinal wall and intestinal villi of all segments in all experimental groups (Fig. 1A–C). The histological appearance of the intestinal villi showed a significant enrichment with supplemented *S. cerevisiae* or *L. bulgaricus* (Fig. 1B2–C4). Interestingly, *L. bulgaricus* gave a slightly better histomorphometry of the intestine than *S. cerevisiae* without significant differences from the combination of both supplemented elements.

**Figure 2:**
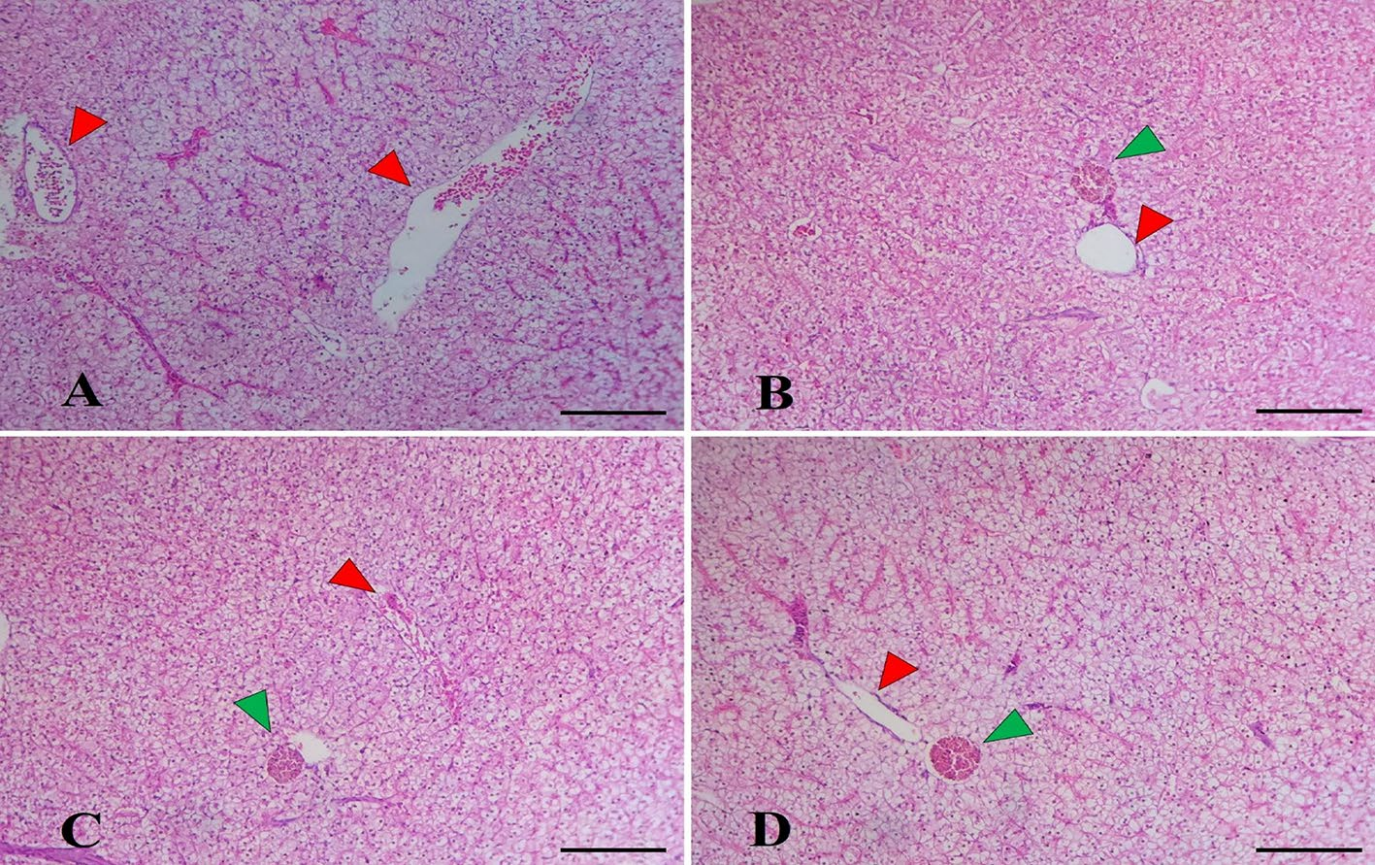
The photograph shows the histological structure of *M. capito* liver in the control group (**A**) as well as other treated groups by *S. cerevisiae* (**B**), *L. bulgaricus* (**C**), and their combination (**D**). The construction of the liver in the control fish presented a normal appearance of hepatocytes (**A**), arranged in hepatic cords that were separated by slightly congested blood sinusoids and central veins. *S. cerevisiae* and *L. bulgaricus*—supplemented groups prompted enhanced hepatic parenchyma with increased glycogen storage, and melanomacrophage centers (green arrowhead), particularly in the mixture group. Stain H&E. Bar 100 µm.

The histopathological examination of the liver in the control group revealed normal hepatic parenchyma and intact hepatocytes arranged in hepatic cords that were separated by slightly congested blood sinusoids and central veins (Fig. 2A). *L. bulgaricus*—supplemented groups as well as *S. cerevisiae* with *L. bulgaricus* (Fig. 2C,D) groups showed more improved construction of the hepatic parenchyma represented in increased glycogen storage with pigmented melanomacrophage centers near the hepatic central veins while *S. cerevisiae*—supplemented group (Fig. 2B) showed less improvement.

## Discussion

A mounting body of research has underscored the capacity of probiotics to enhance growth performance, feed utilization, proximate body composition, antioxidant activity, and hepatic health^11,38^. These beneficial outcomes are often attributed to the administration of single-species probiotic treatments, as elucidated in numerous studies^16,22^. However, scant attention has been devoted to investigating the benefits arising from the concurrent introduction of distinct probiotic species into fish diets^39,40^. It has been hypothesized that a multispecies probiotic combination could potentially offer more advantages compared to single-strain probiotics when administered to fish^24^. This hypothesis is thought to stem from synergistic interactions among probiotic strains, as supported by studies conducted by Abdel-Latif et al.^24^ and Wang et al.^41^.

The growth performance and nutritional outcomes observed in this study indicate the potential benefits of probiotic supplementation in hapa-reared *M. capito*. These findings follow previous studies examining the effects of probiotics on various fish species such as red sea bream (*Pagrus major*)^42^, gilthead sea bream (*Sparus aurata*)^43^, and olive flounder (*Paralichthys olivaceus*)^44^. This enhancement in growth parameters can be attributed to the probiotics’ ability to improve nutrient utilization and digestion efficiency, as reported in previous studies on fish^45,46^. PER and PPV are important indicators of the nutritional value of the diet, and their significant improvement in the combination group suggests that *S. cerevisiae* and *L. bulgaricus* supplementation enhances the utilization of dietary protein. This aligns with research on other fish species, where probiotics have been shown to enhance protein utilization and improve the overall nutritional quality of the diet^4^. The elevated E.R. value in the combination group further supports the notion that probiotic supplementation can optimize protein utilization in *M. capito*. To the best of our knowledge, this study marks the inaugural instance of dietary supplementation with *S. cerevisiae* and/or *L. bulgaricus* yielding improvements in the growth and feed utilization of *M. capito*. The fish survival remained consistently high across all treatment groups at 100%. This finding suggests that the probiotic supplements did not negatively impact fish. This aligns with previous studies that have reported the safety and non-toxic nature of probiotic supplementation in fish^47–49^.

In our current investigation, the CP and CL levels were increased in the whole body of fish-fed diets supplemented with *S. cerevisiae* and/or *L. bulgaricus*. These findings suggest that these probiotics have led to enhanced utilization of both protein and lipids. These results are consistent with prior research, as proposed by Dawood et al.^50^ and Mohapatra et al.^51^.

Probiotics have a potential efficacy in diminishing oxidative stress among aquatic organisms, including fish and shellfish^52,53^. When animals are subjected to stress, it can trigger an increase in the levels of reactive oxygen species (ROS). These ROS play a vital role as chemical signaling molecules within the organism. However, oxidative stress occurs when there is an imbalance between ROS/RNS (reactive nitrogen species) and antioxidants, leading to cell damage through processes such as lipid peroxidation. In response, the fish’s antioxidant defense system becomes activated, combatting the generated free radicals by releasing antioxidant enzymes like SOD, CAT, and GPX. These antioxidant enzymes play pivotal roles in mitigating oxidative stress in fish. These enzymes function to endorse the regular redox equilibrium^54,55^. The results of this study reveal noteworthy variations in antioxidant enzyme activity and oxidative stress levels across the investigated groups, shedding light on the potential benefits of concomitant supplementation with two distinct probiotics. When compared to the control group, both *S. cerevisiae* and *L. bulgaricus* individually demonstrated an increase in the activities of SOD, CAT, and GPX. Notably, the combination group exhibited the most remarkable enhancement in these enzymatic activities. These findings align with previous investigations into the antioxidant effects of probiotics (e.g.^56,57^). The TBARS levels, as an indicator of oxidative stress^58^, followed an opposing trend. The decrease of TBARS in probiotic groups suggests that the combination of *S. cerevisiae* and *L. bulgaricus* exerts a potent antioxidative effect, potentially mitigating oxidative stress-induced damage more effectively than individual probiotic supplementation. Previous research also provides supporting evidence for the role of probiotics possessing antioxidant properties in enhancing intracellular antioxidant enzymes. For instance, Abarike et al.^59^ observed increased SOD and GPX activity in the serum of *Labeo rohita* and *Oreochromis mossambicus* when fed a diet containing 10^8^ CFU/g of *L. plantarum* and 10^7^ CFU/g of *Bacillus licheniformis*, respectively. It was proposed that these probiotics exert their antioxidant effects through mechanisms such as ion chelation, reduction of reactive oxygen metabolites, prevention of oxidant compound production, reduction of ascorbate autoxidation, and scavenging of ROS, as suggested by Amaretti et al.^60^ and Naderi Farsani et al.^57^. Probiotics exert antioxidant effects and enhance fish health through a multifaceted approach involving modulation of gut microbiota, production of antioxidant compounds, reduction of inflammation, enhancement of immune function, improvement of antioxidant enzyme activity, regulation of gut barrier function, detoxification of metabolic byproducts, and enhanced nutrient absorption^61^. These mechanisms collectively contribute to mitigating oxidative stress and promoting overall fish health. Probiotics enhance the diversity and balance of gut microbiota, leading to improved gut health and nutrient absorption^62^. Additionally, certain strains of probiotics produce antioxidant compounds such as glutathione, superoxide dismutase, and catalase, which scavenge free radicals and reduce oxidative stress^63^. Furthermore, probiotics modulate immune responses, reduce inflammation, enhance antioxidant enzyme activity, and regulate gut barrier function, collectively contributing to improved fish health^64,65^. Nonetheless, additional research is warranted to elucidate the underlying mechanisms and evaluate the effects of these probiotics under induced stress conditions, such as overcrowding or transportation. Conducting pro-oxidant challenge studies would allow for a comprehensive assessment of their impact in such scenarios.

The maturation and functionality of internal organs, particularly the intestine and liver, exert a profound impact on the growth of fish, bolstering their resilience against diseases and stressors while enhancing their efficiency in utilizing feed resources^66^. In the present study, the histological analysis of *M. capito* treated with a combination of probiotics exhibited notable improvements in both the intestinal and hepatic tissues compared to the control group. The histological examination of the *M. capito* intestine across all experimental groups revealed that the intestinal wall and villi remained structurally intact. This finding suggests that the dietary supplements, whether *S. cerevisiae* or *L. bulgaricus*, did not cause any significant damage to the intestinal structure. Both supplements appeared to enrich the histomorphometry of the intestinal villi. Previous research has highlighted the beneficial effects of probiotics such as *L. bulgaricus* on gut health. Probiotics can enhance the integrity of the intestinal mucosal barrier, improve the composition of gut microbiota, and promote the growth of beneficial enterocytes and goblet cells^66–68^. The enrichment observed in this study aligns with these known effects of probiotics. Interestingly, the combination of both *S. cerevisiae* and *L. bulgaricus* showed a similar effect on histomorphometry compared to *L. bulgaricus* alone, implying that *L. bulgaricus* may play a predominant role in enhancing intestinal structure. It is essential to note that while the histological structure appeared to improve with these supplements, additional functional studies would be required to ascertain their impact on nutrient absorption and overall gut health.

The groups supplemented with *L. bulgaricus* or the combination of *S. cerevisiae* and *L. bulgaricus* demonstrated an improvement in the construction of hepatic parenchyma. This was characterized by increased glycogen storage and the presence of pigmented melanomacrophage centers near the central veins. Glycogen storage is indicative of the liver’s ability to store glucose and maintain blood sugar levels^69,70^. The presence of melanomacrophage centers suggests an enhanced immune response in the liver^71,72^. This could be attributed to the immunomodulatory properties of probiotics, which have been reported to have anti-inflammatory effects and support liver health^73,74^. Probiotics have been associated with improved liver function, reduced inflammation, and protection against liver diseases^75,76^. Taken together, the histological analysis of the *M. capito* intestine and liver suggests that dietary supplementation with *S. cerevisiae* and *L. bulgaricus* has a positive impact on the structural integrity of these organs. *L. bulgaricus*, in particular, appears to play a significant role in enhancing both intestinal and hepatic histomorphometry. These findings are consistent with existing research on the beneficial effects of probiotics on gut and liver health. However, further studies are needed to elucidate the underlying mechanisms and assess the functional implications of these histological improvements.

## Conclusion

In summary, this study underscores the beneficial impacts of orally administering *S. cerevisiae* and/or *L. bulgaricus* probiotics on various aspects of hapa-reared *M. capito*, including growth, feed utilization, body composition, hepatic antioxidant capacity, and histoarchitectural changes. Notably, the combination of these probiotics in a multispecies formulation exhibited superior efficacy compared to individual supplementation. These findings suggest mixing different probiotics to enhance fish performance and overall health. Nonetheless, further studies are warranted to elucidate the precise mechanisms involved and to assess the long-term effects on fish health and welfare.

### Supplementary Information

Supplementary Table S1.

## Data Availability

The datasets used in this study can be obtained by contacting the corresponding author upon request.
