# Supplementary material for: Evaluation of different probiotics on growth, body composition, antioxidant capacity, and histoarchitecture of Mugil capito
Source: Sci Rep. 2024 Mar 28;14:7379. doi: 10.1038/s41598-024-57489-x (PMC10978984; doi:10.1038/s41598-024-57489-x)
Supplement: Supplementary file 1 — Supplementary Table S1. [file 41598_2024_57489_MOESM1_ESM.doc]

**Table S1 (Supplementary Material).**

The basal diet's constituent components in grams and its proximate chemical composition as a percentage on a dry weight basis.

| **Feed ingredients** | **Control (basal) diet** |
| --- | --- |
| Fish meal | 100 |
| Soybean meal | 280 |
| Corn gluten | 110 |
| Ground corn | 290 |
| Wheat bran | 120 |
| Corn oil | 15 |
| Fish oil | 15 |
| Starch | 30 |
| Di-calcium phosphate ^a^ | 20 |
| Mineral and Vitamin premix ^a^ | 20 |
| Total (g) | 1000 |
| **Proximate chemical analysis (% on a dry weight basis)** | |
| Dry matter | 89.9 |
| Crude protein | 30.2 |
| Crude fiber | 3.4 |
| Ether extract | 7.4 |
| Ash | 6.5 |
| Nitrogen-free extract ^b^ | 52.5 |
| Gross energy (MJ/ kg) ^c^ | 18.9 |

^a^ These ingredients were purchased from AGRI-VET company for Manufacturing Vitamins and Feed Additives (Cairo, 10^th^ of Ramadan City A2, Egypt).

^b^ Nitrogen-free extract was calculated as 100 − (crude protein + ether extract + Crude fiber + Ash).

^c^ Gross energy was calculated based on the values of values for proteins, lipids, and carbohydrates as 23.7, 38.7 and 16.9 KJ/g respectively.
